# Adaptability and Stability of Faba Bean (*Vicia faba* L.) Accessions under Diverse Environments and Herbicide Treatments

**DOI:** 10.3390/plants11030251

**Published:** 2022-01-19

**Authors:** Lynn Abou-Khater, Fouad Maalouf, Abdulqader Jighly, Diego Rubiales, Shiv Kumar

**Affiliations:** 1 International Center for Agricultural Research in the Dry Areas (ICARDA), Beirut 114/5055, Lebanon; f.maalouf@cgiar.org; 2 Agriculture Victoria, Victoria 3083, Australia; abdulqader.jighly@agriculture.vic.gov.au; 3 Institute for Sustainable Agriculture, CSIC, 14004 Córdoba, Spain; diego.rubiales@ias.csic.es; 4 ICARDA, Biodiversity & Integrated Gene Management Program, Rabat 10112, Morocco; sk.agrawal@cgiar.org

**Keywords:** faba bean, herbicide tolerance, G × E interaction, stability parameters, GGE biplot

## Abstract

The adaptability and stability of 37 faba bean (*Vicia faba* L.) accessions with different levels of tolerance to metribuzin or imazethapyr was assessed across 12 season–location–herbicide experiments. Significant Genotype x environment (GE) interaction was found for the days to flowering (DFLR), plant height (PLHT) and grain yield (GY). Performance and stability of the accessions regarding PLHT and GY were assessed using four different stability parameters: cultivar superiority, static stability, Wricke’s eco-valence and Finlay and Wilkinson’s regression model. The stability parameters ranked these genotypes differently suggesting that PLHT and GY stability should be assessed not only on a single or a few stability parameters but on a combination of them. GGE biplot analysis indicated that the environments representing metribuzin treatment at Marchouch 2014–2015 and the non-treated treatment at Terbol 2018–2019 are the ideal environments for evaluating faba bean genotypes. GGE biplots showed herbicide tolerant accession IG12983 with simultaneous average PLHT, GY and stability across the environments. The performance of other tolerant accessions, namely IG13945, IG13906, IG106453, FB2648, and FB1216 was less stable but superior under specific mega environments. Therefore, utilizing these accessions in faba bean breeding programs would help broaden the adaptability to diverse locations–season–herbicide treatments.

## 1. Introduction

Faba bean (*Vicia faba* L.) was domesticated 10,000 years Before Christ (BC) in the Near East where archeological findings of domestic [1] and wild specimens were discovered [2]. Today, faba bean is considered the fourth most important cool season food legume after chickpea, lentils, and peas as it is grown on 2.57 million ha area with a total production of 5.4 million tons in 2019 [3]. This crop plays critical role in supporting nutritional and food security and enhancing soil structure in many countries, including China, Egypt, Ethiopia, United Kingdom, Australia, France, Sudan, and Morocco [3]. Faba bean production has increased 2% annually over the past three decades while global area remains stagnant.

Faba bean is affected by several biotic and abiotic stresses including parasitic and non-parasitic weeds. Among annual weeds, broadleaved and grass species like *Anthemis arvensis* L., *Chenopodium album* L., *Convolvulus arvensis* L., *Sinapis arvensis* L. and *Avena sterilis* L. compete with faba bean crop [4,5]. Parasitic weeds like *Orobanche crenata* L. and *Cuscuta sativa* L. also severely affect faba bean in many production areas [6,7,8]. An integrated weed management that employs a variety of chemical and non-chemical methods is an effective way of minimizing the losses caused by weeds. The development of resistant cultivars to multiple herbicides with different modes of action [9,10,11] is still the most economical and environmentally friendly control strategy to reduce the cost of the weed control practices, and avoid herbicides injury to the crop and herbicide resistance of some weeds [9,12,13]. Research conducted at ICARDA [9,14] resulted in the identification of accessions that tolerate metribuzin and/or imazethapyr herbicides. However, demonstrating the adaptability of these accessions to a wide range of environments can increase their economic value as climate change is expected to reduce the production of faba bean in many regions. Therefore, there is a need to study the yield stability of these accessions under different environmental conditions.

Grain yield is a very complex trait which is strongly influenced by genotype (G), environment (E) and genotype x environment (GE) interaction [15,16]. GE interaction is of major importance for breeders, given that it reduces the association between phenotypic and genotypic values across environments [15,17]. It also affects the identification of relevant test environments, the allocation of resources within a breeding program and the choice of germplasm and breeding strategy [18]. GE interaction is a challenge in the case of legume breeding as previous studies have suggested a high proportion of variance due to environment (E) and GE interaction on the expression of grain yield in pulse crops including faba bean [19,20].

Environmental variation has a major effect on the variation of yield (up to 80% or higher) [21] in developed pure lines with narrow genetic base, but genotypes and GE interaction are more relevant for germplasm evaluation and selection and they must be considered simultaneously when selecting a genotype; in other words, an ideal genotype should have both high mean yield performance and high stability across environments [22,23].

The major objective of this research was to assess yield stability of selected faba bean accessions for herbicide tolerance under different environments with combined effect of herbicide treatment, location, and season. The second objective was to identify mega-environments and the best environments to screen faba bean for herbicide tolerance.

## 2. Results

### 2.1. Phenological Traits

Combined analysis of variance showed significant differences among the 37 accessions across the 12 environments for days to flowering (DFLR) and days to maturity (DMAT) reflecting the presence of genotypic variability for both traits. In addition, the interaction between Genotype x Herbicide treatment was highly significant (Table 1) indicating that the studied genotypes behaved differently under different herbicide treatments for DFLR and DMAT in the different environments. Very high values of narrow sense heritability (0.97 and 0.99) for both DFLR and DMAT were estimated under different herbicide treatments and across different locations-seasons (Table 1).

The analysis of variance conducted for each environment showed significant differences (*p* < 0.001) among accessions in all environments for DFLR except in Marchouch 2016/2017-imazethapyr (environment E), where the season was dry and warm. The flowering time was earlier in the environments C, F, I and L where no herbicide treatment was applied at both locations and in different cropping seasons. Means and ranges of DFLR are presented in Table 2. This varied among accessions from 39 days at Marchouch 2016/2017 without herbicide treatments (environment F) to 53 days after sowing (DAS) at Marchouch 2016/2017-imazethapyr treatment (environment E) and the widest range was observed in Terbol 2015/2016-imazethapyr treatment (environment H) where it varied from 93 to 131 DAS.

The analysis of variance revealed significant differences (*p* < 0.001) among accessions in all environments for days to maturity (DMAT) except in environments E, H and K where they were treated with 75 g ai/ha of imazethapyr in Marchouch 2016/2017 and Terbol 2016/2017 and 2018/2019 seasons.

The maturity time was delayed in trials treated by both metribuzin and imazethapyr herbicides over season–locations than those with no herbicide application (Table 2). The narrowest range of maturity time was observed in Terbol-2015/2016 with no herbicide treatment (environment I) where it varied between 165 and 171 DAS, and the widest range was observed in Marchouch 2014/2015 with no herbicide treatment (environment C) where it varied from 131 to 147 DAS (Table 2).

### 2.2. Plant Height

**Figure 1:**
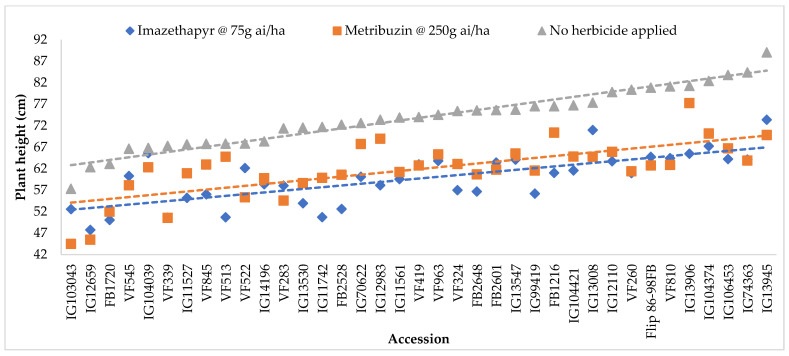
Mean plant height (cm) of studied accessions of faba bean across seasons–locations under imazethapyr, metribuzin and no herbicide application treatments (SE = 5.149).

Combined analysis of variance showed that plant height varied significantly among genotypes and herbicide treatments, but no significant GE and Genotype × Herbicide Treatment interactions were observed across environments (Table 1, Figure 1). However, significant differences among genotypes for plant height were detected in all environments except in Marchouch during 2016/17-imazethapyr treatment (environment E) where severe terminal drought occurred. Narrow sense heritability of plant height was relatively high (0.60) indicating replicability of the traits among accessions in different herbicide treatments and across different locations-seasons (Table 1).

h^2^_PLHT varied between 0.01 in Terbol 2015/2016-no herbicide treatment (environment I) and Terbol 2018/2019-metribuzin treatment (environment J) and 0.95 in Marchouch 2014/2015-metribuzin treatment (environment A) (Table 3). However, significant GE interaction was also observed for plant height indicating that the genotypes responded differently in different seasons and locations (Table 1).

All tested accessions had lower plant height under metribuzin and imazethapyr than under no herbicide application (Table 2). Average plant height varied from 18 cm in Terbol-2015/2016–metribuzin treatment (environment G) and 112 cm in Terbol-2018/2019-no herbicide treatment (environment L). Figure 1 showed that non-treated plants tended to have the highest height, followed by the plants treated with metribuzin and then by those treated with imazethapyr. The plant height of accession IG104039 classified previously as tolerant to both herbicides did not differ significantly under metribuzin or imazethapyr treatments across all environments, and the plant height of accession VF513 classified previously as tolerant to metribuzin did not differ significantly under metribuzin treatment across environments (Figure 1).

As significant GE interactions were detected for plant height, the stability parameters were assessed to determine the specific response of the tested accessions using the four stability parameters, namely cultivar superiority, static stability, Wricke’s eco-valence [24] and Finlay and Wilkinson [25] stability parameter.

The rankings of the accessions based on the plant height stability are presented in Table 4. Considering the cultivar superiority and the ability of the genotypes to have a mean plant height above average across environments, IG13945 had the best plant height performance across environments. As for the ability of the genotypes to maintain a stable performance across environments, FB2601 was considered the most stable based on the static stability parameter, VF845 based on Wricke’s eco-valence, as it had the lowest eco-valence value, and IG12659 based on Finaly and Wilkinson stability parameter, as it received the lowest values for these parameters.

Among the genotypes that had plant height above average and performed well in all environments, only four FB2601, IG104374, IG104421 and Flip86-98FB had small fluctuation across environments and were identified as stable by two of the four stability parameters. Among these genotypes classified earlier as moderately tolerant/tolerant to both herbicides, IG104374, IG104421 and Flip86-98FB were identified as stable by Wricke’s eco-valence and FB2601 and Flip 86-98FB by the static stability parameter. Wricke’s eco-valence followed by the static stability parameter was effective in simultaneously selecting stable genotypes with high plant height unlike the Finlay and Wilkinson’s stability parameters which, in this study, identified mostly genotypes with low plant height as being the most stable.

The correlations among the stability parameters are shown in Table 5 The correlation coefficient varied between −0.4 and 0.9 indicating an inconsistency in the classification between the parameters. Significant negative correlation (−0.4) was observed between Cultivar superiority and Finley and Wilkinson’s parameter and highly significant and positive correlation was observed between Finley and Wilkinson’s parameter and Static stability (0.9) and between Wricke’s eco-valence and Static stability (0.7). However, even with strong correlation between methods, genotype ranking can be different. FB2601, Flip86-98 and IG70622 had the most stable plant height as they ranked among the 10 most stable accessions by different stability parameters.

### 2.3. Grain Yield

Combined analysis of variance revealed significant variation among genotypes and herbicide treatments (Table 1). Significant GE and Genotype × Herbicide treatment interactions were also observed for grain yield indicating that the genotypes responded differently to different environments characterized by different herbicide treatments, seasons, and years (Table 1). The h^2^_GY was average (0.40) ranging from nearly zero in three environments of Marchouch 2015/2016 (environment D, E, F) and in Terbol 2018/2019-metribuzin treatment (environment J) and Terbol 2018/2019-no herbicide treatment (environment L) to 0.5 in three environments of Marchouch 2014/2015 (environment A, B, C) (Table 3). This indicates diverse response of each accession to the different herbicide treatments and across the different locations-seasons combinations.

**Figure 2:**
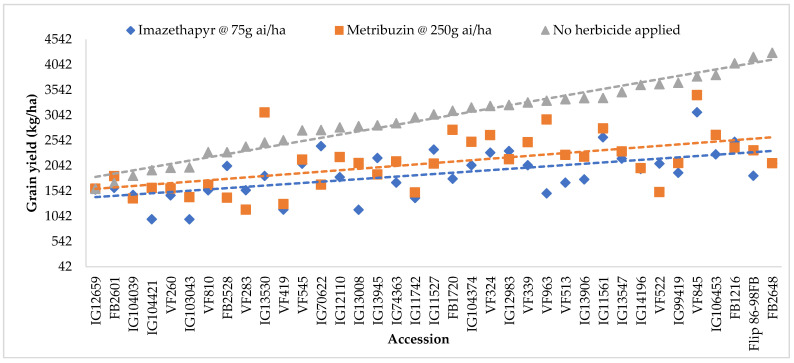
Mean grain yield (kg/ha) of studied accessions across seasons–locations under imazethapyr, metribuzin and no herbicide application treatments (SE = 38.76).

In addition, Genotype × Herbicide Treatment and GE interactions were highly significant indicating that the accessions performed differently under different herbicide treatments and in different locations and seasons (Table 1). Furthermore, Figure 2 indicated that some accessions yielded more under metribuzin and imazethapyr than under no herbicide application. This is also shown by the drown trends of each herbicide applications.

The average grain yield was lower in environments treated with herbicides than in environments with no herbicide treatment at both locations in all seasons (Table 2). Figure 2 showed that the non-treated plants tend to have the highest grain yield, followed by the plants treated with metribuzin and then by those treated with imazethapyr. However, the tolerant and moderately tolerant accessions (FB2601 and IG13530) to both herbicides yielded more under metribuzin than under no herbicide treatment across environments, and the tolerant accessions (IG70622 and FB2528) yielded similarly under imazethapyr treatment and under no herbicide treatment across environments.

As significant GE interactions were detected for grain yield, the grain yield stability was assessed based on the same parameters used to evaluate the plant height stability as presented in Table 6. Considering the cultivar superiority and the ability of a genotype to have an above average mean performance across environments, VF845 had the best grain yield performance in all environments. As for the ability of the genotypes to maintain a stable performance across environments FB2528 was considered the most stable based on the static stability parameter, VF810 based on Wricke’s eco-valence, as it had the lowest eco-valence value, and IG103043 based on Finlay and Wilkinson stability parameter, as they received the lowest values of these parameters.

Among the genotypes that had grain yield above average in all environments, IG12983, which was tolerant to metribuzin and imazethapyr, was the only one that was able to maintain a stable performance across environments; it was identified as stable by Wricke’s eco-valence. Wricke’s eco-valence was the only effective parameter in identifying a stable and high yielding genotype, unlike the static stability and Finlay and Wilkinson parameters that identified mostly genotypes with low grain yield as being the most stable.

The correlation between the stability parameters is shown in Table 5. The correlation coefficient varied between −0.6 and 0.6. This result indicates inconsistency in the classification between the parameters. Highly significant and negative correlation (−0.6) was observed between static stability and Finley and Wilkinson’s parameter and highly significant and positive correlation was observed between static stability and Wricke’s eco-valence (0.6). However, even with strong correlation between methods, genotype ranking can be different between the methods. IG11527 and VF810 had the most stable grain yield as they ranked among the 10 most stable accessions by different stability parameters at the same time.

### 2.4. GGE Analysis

**Figure 3:**
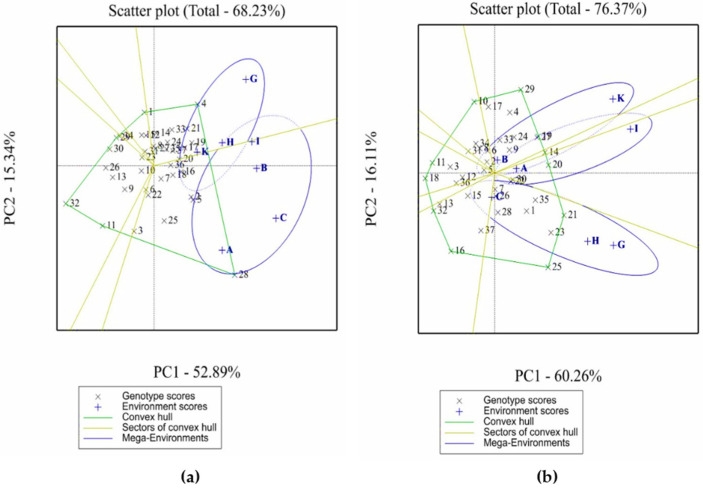
GGE-biplot showing the performance of each faba bean genotype in each environment and the “who-win-where” pattern of genotypes and environments for plant height (**a**) and grain yield (**b**).

The GGE-biplots presented in Figure 3 accounted for 68.23% and 76.37% of the total variability for the plant height and grain yield, respectively. The environments with low narrow sense heritability values were excluded from the GGE biplot analysis as most of the variations observed are not genetic and might be related to the environmental conditions. The values of narrow sense heritability obtained in the present study shows that the terminal drought at Marchouch 2014/2015 and high rainfall at Terbol 2018/2019 might have led to low values and therefore the corresponding environments (D, E, F, J, K and L) were excluded from the analysis.

#### 2.4.1. Evaluation of Test Environments

GGE-biplot provides a summary of the relationship between test environments. Two environments are positively correlated if the angle between their vectors is less than 90° [26]. Based on this, the plant height biplot (Figure 3a) showed positive and high correlation between two imazethapyr @75 g ai/ha treatments of Terbol 2015/2016 and Terbol 2018/2019 (environments H and K) and the non-treated treatment of Terbol 2015/2016 (environment I). However, there was low correlation between environments A (metribuzin 250 g ai/ha-Marchouch 2014/2015) and G (metribuzin 250 g ai/ha-Terbol 2016/2017) as the angle between the two corresponding vectors was nearly 90°.

On the other hand, the grain yield biplot (Figure 3b) shows positive and high correlation between the non-treated treatment Terbol 2015/2016 (environments I) and the metribuzin 250 g ai/ha of Marchouch 2014/2015 (A) and between the metribuzin 250 g ai/ha of Terbol 2015/2016 (G) and the imazethapyr 75 g ai/ha of Terbol 2015/2016 (H). However, the correlation between imazethapyr 75 g ai/ha (B) and non-treated treatment of Marchouch 2014/2015 (C) indicated no association.

GGE biplots (Figure 3) also provide information about the discriminating ability of each test environment for plant height and grain yield. Figure 3a shows that, among the test environments, 250 g ai/ha metribuzin of Terbol 2015/2016 (environment A) and non-treated treatments of Terbol 2015/2016 (C) and Marchouch 2014/2015 (G) had the longest vector and hence were highly discriminating for plant height evaluation. On the other hand, metribuzin 250 g ai/ha (G) and non-treated treatment of Terbol-2015/2016 (I) and imazethapyr 75 g ai/ha treatment of Terbol-2018/2019 (K) were highly discriminating for grain yield evaluation (Figure 3b). Moreover, the least discriminating environments are those having the shortest vectors. Based on this, imazethapyr 75 g ai/ha treatments of Terbol 2015/2016 and Terbol 2018/2019 were identified as the least discriminating environments for plant height evaluation while three environments of Marchouch 2014/2015 were identified as the least discriminating environments for grain yield evaluation.

#### 2.4.2. Identification of Mega-Environments and Specific Adapted Accessions

The plant height GGE biplot (Figure 3a) was divided into 6 sections where the 12 environments fell into two of these, and accordingly two mega-environments were identified. The first mega-environment contained three environments of Marchouch 2014/2015 (environments A, B and C), and the non-treated treatment of Terbol 2015/2016 (I). The second mega-environment includes environments G (metribuzin 250 g ai/ha treatment-Terbol 2015/2016), H (imazethapyr 75 g ai/ha-Terbol 2015/2016) and K (imazethapyr 75 ai Terbol 2018/2019). IG13945 (28) and IG13906 (4), which are moderately tolerant or tolerant to metribuzin and imazethapyr, were the tallest genotypes in the first and in the second mega-environments, respectively. The GGE biplot for grain yield (Figure 3b) was divided into 6 sections where the 12 environments fell into three sections and accordingly three mega-environments were identified. The first mega-environment had imazethapyr 75 g ai/ha of Marchouch 2014/2015 (B) and Terbol 2018/2019 (K); the second one had 250 g ai/ha metribuzin of Marchouch 2014/2015 (A) and non-treated treatment of Terbol 2015/2016 (I) while the third mega-environment had metribuzin 250 g ai/ha (C) and imazethapyr 75 g ai/ha (G) of Terbol 2015/2016 and non-treated treatment of Marchouch 2014/2015 (H). IG106453 (19) had the highest yield in the first mega environment, FB2648(14) in the second and FB1216 (21) in the third mega environments.

#### 2.4.3. Performance of Tested Accessions

**Figure 4:**
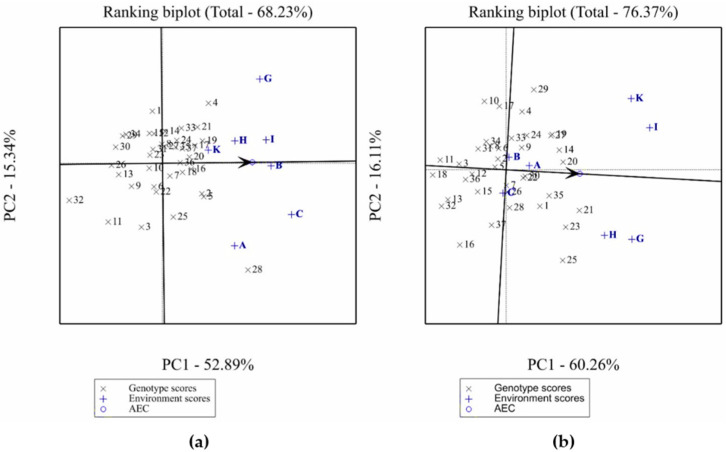
Ranking plot of the GGE showing the performance and stability of the faba bean genotypes for plant height (**a**) and seed yield (**b**).

Figure 4a,b show the ranking of genotypes based on plant height and grain yield performance and stability in 12 environments. The mean yield performance and stability of genotypes were evaluated using an average tester axis (ATA) that passes through the origin [27,28,29]. Based on Figure 4a, 17 accessions were shorter than the average plant height across the 7 environments as they are located on the left side of ATA. The other 20 accessions were taller than the average across the 7 environments as they are located on the right side of ATA; 7 of them were tolerant to both herbicides’ treatments across environments.

Figure 4a shows that among the accessions tolerant to both herbicides with plant height above the average, FB2601 (16), IG104421 (18) and IG12983 (35) were the most stable as they had the shortest projection to the ATA. On the other hand, despite being moderately tolerant/tolerant to metribuzin and imazethapyr with good plant height performance across seven environments, IG13945 (28) was the least stable among all the accessions given that it had the longest projection to the ATA.

Regarding grain yield performance, Figure 4b shows that 18 accessions yielded less than the average grain yield across the seven environments as they are located on the left side of the ATA. The remaining 19 accessions yielded more than the average grain yield as they were located on the right side of ATA; 6 of them were tolerant to both herbicides’ treatments across environments. Among the accessions that yielded more than the average grain yield and are tolerant to both herbicides, FB2648 (14), IG12983 (35) and VF522 (9) were the most stable as they had the shortest projection to the ATA. Hence, based on Figure 4a,b, the metribuzin and imazethapyr tolerant accession IG12983 (35) was considered a superior genotype in terms of plant height and grain yield as it showed a good and stable performance for both traits across environments. Furthermore, the following accessions VF963 (27), FB2648 (14), and IG70622 (33) that are tolerant to both herbicides performed well and were moderately stable across the 7 environments.

## 3. Discussion

Multi-environment trials are an integral part of the breeding pipeline to better understand the performance of tested accessions under a wide range of environmental conditions and therefore breeders are able to characterize the mega environments and identify cultivars adapted to specific environments or with broad adaptability [20,30]. In this study, faba bean accessions were evaluated in a wide range of environmental conditions created by different site–season–herbicide treatment combinations.

Faba bean accessions flowered and matured earlier in the environments with no herbicide treatment than in the environments with metribuzin or imazethapyr treatments in all sites and seasons. Past studies also reported a delay in flowering and maturity time of different legume crops treated with metribuzin or imazethapyr [31,32,33,34,35]. This delay might be the result of the growth inhibition of the crops amid their treatment with herbicide [36,37,38]. Faba bean accessions flowered and matured earlier in Marchouch than in Terbol. This might be attributed to cooler and more rainy weather in Terbol as compared to Marchouch. Past studies also reported decline in crop duration under heat and drought conditions in faba bean [39,40], lentil [41,42], chickpea [43], and common bean [44]. The earlier onset of flowering and maturity was observed in the non-treated environment of Marchouch 2016–2017 season; this was expected as it resulted from a combination of an exceptional warm and dry season and no herbicide treatment.

Plant height and grain yield were higher in the environments with no herbicide treatment than in the environments with metribuzin or imazethapyr treatments in all sites and seasons. Several studies also reported reduction in plant height and grain yield of accessions treated by herbicides in chickpea [34,45] and lentil [31,46]. This reduction might be due to the growth and photosynthesis inhibition caused by both metribuzin and imazethapyr [36,38,47]. Plant height and grain yield were higher in Terbol clustered under high rainfall than in Marchouch with low rainfall conditions. The highest average plant height and grain yield were recorded in the experiments with no herbicide treatments at Terbol 2018–2019; this was expected as it resulted from a combination of lowest temperatures, highest rainfall conditions and no herbicide application.

The heritability estimates are effective when combining data from diverse environments as the phenotypic value used to estimate the heritability is the mean value obtained across experiments and replicates [48]. In our study, heritability estimated for grain yield was highly affected by the stress environments followed by those estimated for plant height, and days to flowering and maturity. Similar observations were reported for heritability in faba bean by Toker [15]. Mohamed [49], Abdelmula et al. [50], Ceccarelli [51], and Atlin and Frey [52] concluded that lower heritability was expected in low-yielding environments. Therefore, the selection of faba bean genotypes is best done under optimum environments that are less likely to encounter stress-periods. Furthermore, moderate to high values of narrow sense heritability reported in the present study are important because the response to selection depends on the additive genetic variance captured by the narrow-sense heritability [53] and therefore they make a good basis for further genetic analysis and allow for true replication of a genotype in and across multiple environments [48,54].

Breeding programs focus on the evaluation of the performance and stability of accessions that have traits of economic importance under diverse environments. The stability analysis conducted in the present study allowed the identification of stable and high yielding genotypes across different environmental conditions. A stable genotype should have an above average and stable performance across environments [22,23]. The various stability parameters used in this study ranked plant height and grain yield of genotypes differently at different test environments. The inconsistency in ranking of cultivar superiority, Finlay and Wilkinson and Wricke’s eco-valence indices were also reported in faba bean [21], pearl millet [55] and maize [56]. Our results agree with the results reported by Mustapha and Bakari [57] who observed no similarity between static and cultivar superiority but are contrary to the ones reported by Dehghani et al. [58] who observed similarity between Finlay and Wilkinson and cultivar superiority when ranking lentil genotypes.

Our results suggest that some genotypes had stable plant height and grain yield performance based on more than one parameter, but their rankings differed with each parameter. This implies that the comparisons may greatly depend on the parameter used as also observed by Milioli et al. [59] and Westcott [60] and thus more than one parameter should be used to characterize and explore performance of genotypes across environments and enable more reliable selection and recommendation of genotypes [61].

Our results also suggest that the selection for genotypic performance stability based on the static stability, Wricke’s eco-valence and Finlay-Wilkinson parameters favor genotypes having plant height and grain yield lower than the population averages. Similarly, Temesgen et al. [21] and Fikere et al. [62] also reported that low-yielding faba bean and lentil genotypes were more stable than high-yielding ones.

Static stability was highly correlated with two other stability parameters for both plant height and grain yield. Seife and Tena [63] found that Wricke’s eco-valence was positively correlated with all stability parameters. However, selecting genotypes based on this method exclusively may not be suitable to identify faba bean accessions that are high-yielding and stable. The use of the Finlay and Wilkinson parameter and Static stability as a selection tool would favor superior and stable genotypes for plant height and grain yield, respectively. Temesgen et al. [21] identified high yielded genotypes that show static stability despite the finding that both high yield and static stability rarely occur in multi-location trials. The classification of low yielding genotypes as stable and high yielding genotypes as unstable by the different stability parameters might be due to the type of accessions evaluated. In the present study, the evaluated accessions are pure lines that have narrow genetic base, narrow adaptability and generally are low yielding and unstable due to homozygosity [64,65].

Plant breeders routinely conduct GGE biplot analysis of multi-environment trials to identify ideal test locations, to reduce the cost of breeding and testing strategies, and to identify genotypes that are widely or specifically adapted. The partitioning of the total sum of squares through GGE biplots obtained in our study shows that there were differential plant height and grain yield performances of faba bean genotypes across environments and consequently a high GE interaction. This interaction could reduce the accuracy of genotype evaluation and selection process [66]. Many GGE studies have been carried out in faba bean and other crops but none of them covers the effect of herbicide treatments. The present study employed a GGE biplot to analyze data from multi-location trials carried out across different locations and under different herbicide treatments over three years. Herbicide application is greatly influenced by weather conditions [67,68,69] and therefore evaluation of the environments and genotypes with herbicide treatment is pertinent to identify genotypes with stable herbicide tolerance.

The GGE biplot was used to evaluate the test environments. An environment is considered ideal for genotype testing when it discriminates the genotypes and represents the environments [16]. The presence of correlation between two environments means that similar information about the genotype performance is derived from them [23] and therefore could be an option to reduce the number of test environments and, as a result, to establish a cost-effective breeding program. The correlations observed in our study between two environments are reliable as both plant height and grain yield biplots accounted for more than 60% of the total variation [29]. Yang et al. [70] claimed that a GGE biplot is considered useful if the two PCs account for more than 60% of the (G+GE) variability. As the GGE biplot that included all the environments accounted for only a small percentage (less than 60%) of the total variability, the patterns obtained were considered less useful and a more reliable and informative GGE biplot was obtained after excluding the low heritable environments.

According to Yan and Tinker [26], the test environments that are less discriminating provide little information on the genotype differences and should not be used as test environments. Hence, in this study among the seven test environments, environment G (metribuzin treatment of Terbol 2015/2016) is the ideal environment for plant height evaluation of genotypes as it is highly correlated with other environments (K, I, H, and B) and is highly discriminating. On the other hand, the ideal environment for evaluating grain yield of faba bean genotype is also environment G (metribuzin treatment of Terbol 2015/2016) as it is highly correlated with many other environments (H, A, B and C) and is highly discriminating. Our results suggest that the discriminating ability of environments was highly influenced by the weather conditions as the three environments of Marchouch 2016–2017 where the weather was exceptionally warm and dry were the least discriminating for grain yield evaluation and therefore, when choosing the testing sites for herbicide tolerance it is better to choose a site that is less likely to have a warm and dry spell period during the growing season like Terbol station.

GGE biplot is an effective visual tool for identifying the mega-environment issues and showing the specific adaptation of the genotypes and which cultivar won in which environments [29,71]. A mega-environment is defined as a group of locations that consistently share the same best cultivar(s) [27]. “Which-won-where” plots constructed in the present study grouped the test environments that represent a combination of season–location-herbicide treatment into different mega-environments. However, the grouping did not correspond with the geographic location or herbicide treatment applied; the grouping seemed to be influenced by the weather conditions and the non-repeatability of the winning genotype in the same geographic location or under same herbicide treatments might be the result of the weather fluctuations observed in the same location from one season to another and to the effect that the weather conditions have on the efficiency of herbicide treatments.

To delineate a mega-environment, the consistency of the genotype’s performance and the repeatability of the winning genotype in the same locations are necessary [72]. The reason for not meeting this condition in our study might be because plant height and grain yield biplots couldn’t capture all the GE variation. Mega-environments are homogeneous groups of locations that reduce research costs by enabling fewer representative environments to be selected for genotype evaluation [73]. However, the identification of mega-environments is not easy. Many studies identified mega-environments in faba bean for grain yield and chocolate spot (*Botrytis fabae*) disease resistance [74], autumn or spring sowing adaptation [20,30] and resistance to Ascochyta blight (*Ascochyta fabae*) [75] and many other studies attempted to define mega-environments in spring wheat [76], sugarcane [77] and rice [78]. Our study is the first attempt to identify mega environments that englobe diverse herbicide treatments.

An ideal genotype is defined as one of the highest yielding across the test environments and is stable in performance [16]. The metribuzin and imazethapyr tolerant accessions which showed a good and stable plant height and grain yield performances in the current study are promising and of great importance for faba bean growers. As plant height is highly correlated with the biological yield [39,79], the accessions showing high and stable plant height performance are very important for the regions where faba bean is grown mainly for animal feeding such as Ethiopia and the Mediterranean countries. The environments evaluated in the present study represented different herbicide treatments applied in different sites and seasons. Hence, the genotypes mentioned are less sensitive to the environmental changes, have high yield, and are tolerant to metribuzin and imazethapyr treatments. Some of the high yielding faba bean genotypes reported in the present study were sensitive to environmental changes. Similar results were also obtained in other crops such as soybean [80,81].

## 4. Materials and Methods

### 4.1. Materials

Thirty-seven faba bean accessions with different level of tolerance to one or both herbicides, metribuzin and imazethapyr, were evaluated in this study. Thirty-six accessions were pure lines derived from single plant selections in three consecutive seasons which represent landraces from 21 countries and one cultivar Elisar (Flip 86-98FB) released in Lebanon was used as check (Table S1). Among them, 14 were tolerant or moderately tolerant to both the herbicides @ 250 g ai/ha metribuzin and 75 g ai/ha imazethapyr, six were tolerant to 250 g ai/ha metribuzin but sensitive to imazethapyr, and 16 were tolerant to 75 g ai/ha imazethapyr but sensitive to metribuzin ([9], ICARDA, unpublished data).

### 4.2. Experiments

A total of 12 experiments combining locations, seasons and herbicide treatments were conducted at Marchouch in Morocco (33.56° N, 6.69° W, 255 m) during the main cropping seasons of 2014/2015, and Terbol in the Bekaa Valley of Lebanon (35.98° N, 33.88° E, 890 m) during 2015/2016, 2016/2017 and 2018/2019 as detailed in Table 7. Each combination of herbicide treatment, location and season was considered as an independent environment. The experiments were conducted in an incomplete block design with two replicates. The plot size was 2 rows with one-meter length with rows spaced at 45 cm and plants spaced at 20 cm within the row. Terbol station is characterized by a cool high rainfall winter and a moderate wet spring. The rainfall recorded during 2015/2016 and 2017/2018 winter cropping seasons were 343 mm and 810.2, respectively. A supplemental irrigation of 30 mm was provided during 2015/2016 to compensate the dry spell periods. Spring 2017/2018 was warmer than normal spring seasons (Table 7). The soil in Terbol station is deep and rich clay loam. Marchouch station is characterized by semi-arid environment, low rainfall and moderate temperature during winter and spring seasons. The annual rainfall recorded during 2014/2015 and 2016/2017 cropping seasons were lower than the mean annual rainfall (396 mm) and the spring season of 2016/2017 was relatively warmer than normal spring seasons. The soil at Marchouch is Vertisol mostly silty clay (Table 7).

The experiments were supplied with 250 kg/ha of granulated NPK (15:15:15) during land preparation. The experiments were sown in late November at Terbol and mid-December at Marchouch and harvested by the end of May at both locations. Necessary phytosanitary and agronomic management practices were applied to ensure a good crop stand: lambda-cyhalothrin @ 40 g ai/ha was sprayed to control sitona weevil (*Sitona lineatus* L.), imidacloprid @ 160 g ai/ha was sprayed to control aphids and azoxystrobin @ 72.8 g ai/ha and difenoconazole @ 45.6 g ai/ha were spayed alternatively to control foliar diseases. To control weeds in all environments, we applied 1200 g ai/ha of pendimethalin as preemergence treatment in addition to post-emergence herbicides. For post-emergence herbicide treatments, two herbicides, namely Metribuzin @250 g ai/ha and Imazethapyr @75 g ai/ha were sprayed uniformly at the stage of inflorescence emergence-BBCH stage 50 [82,83] in addition to a control untreated treatment where hand weeding was applied to ensure an unbiased evaluation of the performance and stability of the faba bean accessions as they were evaluated under the same conditions except for the herbicide treatment.

### 4.3. Recorded Traits

The following traits were measured based on the faba bean ontology described by Maalouf [84]: days to flowering (DFLR) and days to maturity (DMAT) were recorded on plot basis while grain yield per plant (GY) and plant height (PLHT) were recorded on three random plants from each plot and averaged.

### 4.4. Statistical Analysis

The spatial statistical model was applied for variance analysis using the Automatic Spatial Analysis of incomplete block design modules of GenStat 19 edition [85]. Variation among accessions and treatments was assessed in terms of *p*-values using the Wald statistic, and the best unbiased phenotypic estimates of accessions (A) were estimated with standard error using best linear unbiased prediction values (BLUP) using GenStat software. Narrow-sense heritability for each environment (h^2^) was estimated for the PLHT and GY using the method of residual maximum likelihood (REML) and combined narrow sense heritability (h^2^) was estimated for the traits based on the combined analysis using REML model and Best unbiased estimated values of Genstat 2019.

In our study the environment is defined as the combination of year–locations and herbicide treatments either metribuzin or imazethapyr. The following four stability parameters were assessed using GenStat software by comparing different treatments and environments: cultivar superiority index [61], which refers to the ability of the accession to perform above the mean in different environments, static stability coefficient; [86] which refers to the consistency of accession’s performance across different environments; Wricke’s ecovalence [24], which refers to the contribution of the accession to GE interaction; and the index of Finlay and Wilkinson [25], which refers to the response of an accession to different environments by fitting a regression of the environment means for each accession on the average environmental means.

The GGE biplot is an ideal tool for the analysis of data from multi-environment trials (MET); it considers both G and GE interaction effects and graphically displays GE interaction in a two-way table [87]. GGE biplot allows visual examination of the relationships among the test environments, the performance and stability of the genotypes and the mega-environment analysis to recommend specific genotypes for specific mega-environments [16,20,26].

GGE biplot analyses of tested accessions were conducted using the BLUPs obtained under diverse herbicide treatments at two locations: Terbol which is characterized by high rainfall, and Marchouch by low rainfall. The environments with low narrow sense heritability for both GY and PLHT were excluded from the GGE biplot analysis as most of the variations are related to the environmental conditions.

The relationship between the environments was visualized by drawing a vector that connected each environment to the biplot origin:

- The correlation between two environments was approximated based on the angle between two vectors [26,88]; the smallest the angle between two vectors, the highest is the correlation between the two environments.
- The discriminating ability of the test environments was evaluated based on the length of the vector of each environment; the longer the environment vector, the more the discriminating ability of the environment.

The best genotypes for each environment and the possibility of existence of mega environments were identified using the “who-win-where” visualization [29,87]; the polygon view of a biplot (convex hull) is the best way to visualize the interaction between the genotypes and the environments [16]; each polygon was formed by connecting the genotypes that are farthest from the biplot origin so that all other genotypes are inside the polygon [20]. The perpendicular lines to the sides of the polygon divide the biplot into sectors. Each sector has a vertex genotype. The vertex genotype is the one having the longest vector and is considered the winning genotype. The mega-environment was identified as the group of environments that share the same winning genotypes following Yan and Rajcan [27].

A genotype is considered superior when it has both high mean performance and high stability across the test environments. The mean yield performance and stability of genotypes were evaluated by an average environment coordination (AEC) method [27,28,29]:

- The mean performance of the genotype was graphically evaluated based on the line perpendicular to the average tester axis (ATA) that passes through the origin and separates entries with below-average means from those with above-average means; the genotypes located on the right side of this line are taller or have more yield than the ones located on the left side.
- The stability of the accessions was graphically represented by the projection from the genotype to the ATA; the longer the projection the greater is the GE interaction and therefore the lower the stability of the genotype across environments.

## 5. Conclusions

The present study shows significant Genotype × Environment interaction for grain yield and plant height which highlights the need to select genotypes well adapted to specific environment as well as broadly adapted genotypes. The performance and stability of faba bean genotypes were analyzed using four different stability parameters. These stability parameters showed inconsistency in the ranking of genotypes and showed that different stability parameters tend to favor low yielding genotypes. These parameters may not be appropriate, as both breeders and farmers prefer to adopt genotypes that are high-yielding and at the same time perform consistently across environments. GGE identified IG12983 as a superior genotype in terms of plant height and grain yield as it showed a good and stable performance for both traits across locations, seasons, and herbicide treatments which make it attractive to the farmers as it can provide an effective weed management tool. Some accessions had specific adaptation to at least one of the defined mega environments but not to others; IG13945 and IG13906 were specifically adapted to one mega environment in terms of plant height while IG106453, FB2648 and FB1216 were specifically adapted to one mega environment in terms of grain yield. Moreover, some accessions were high yielding but unstable across environments while others were low yielding and stable. To develop superior herbicide tolerant cultivars that are broadly adapted to different mega environments, there is a need to cross the tolerant germplasm identified in the present study with other cultivars adapted to specific environments. These lines could also be crossed with other cultivars to accumulate traits with economical interest in new faba bean varieties. Furthermore, this study suggests conducting herbicide screenings under environments that are less likely to experience drought to avoid the confounding effect of herbicides and drought.

## Figures and Tables

**Table 1:** Combined analysis performed for detecting Wald statistics and differences among faba bean genotypes, treatments, and genotypes x treatment interaction for phenological and agronomic traits across environments.

	df	DFLR	DMAT	PLHT	GY
Genotype × Environment	396	949.1 ***	206	728.8 ***	800.4 ***
Herbicide treatments (T)	2	2.52	4.8	36.56 ***	33.5 ***
Genotypes (G)	36	1859.3 ***	199.2 ***	278.1 ***	268.1 ***
G × T	72	156.5 ***	97.1 *	70.7	125.5 ***
*h^2^*	-	0.97	0.99	0.60	0.40

Df. degree of freedom, DFLR days to flowering, DMAT days to maturity, PLHT plant height, GY grain yield, * *p* < 0.05, *** *p* < 0.001, h^2^ narrow sense heritability.

**Table 2:** Means ± Standard error (SE) and ranges for different traits in the different environments.

Environment	Environment Details	Means	DFLR	DMAT	PLHT (cm)	GY (Kg/ha)
A	Marchouch 2014/2015 treated by metribuzin 250 g ai/ha	Range	ND	ND	28–78	440–5995
		Mean ± SE	ND	ND	55.8 ± 1.5	2344 ± 620
B	Marchouch 2014/2015 treated by imazethapyr 75 g ai/ha	Range	ND	ND	37–82	220–6380
		Mean ± SE	ND	ND	55.1 ± 8.9	1450 ± 1158
C	Marchouch 2014/2015 with no herbicide treatment	Range	78–97	131–147	50–112	333–7333
		Mean ± SE	84.9 ± 3.81	137.9 ± 3.6	72.5 ± 7.5	3545 ± 1360
D	Marchouch-2016/2017 treated by metribuzin 250 g ai/ha	Range	37–47	100–109	32–73	363–3091
		Mean ± SE	41.1 ± 1.6	103.8 ± 1.9	52.1 ± 8.2	1857 ± 392.2
E	Marchouch-2016/2017 treated by imazethapyr 75 g ai/ha	Range	45–53	101–111	28–75	330–2486
		Mean ± SE	47.9 ± 1.6	107 ± 2.26	48.2 ± 9.9	1385 ± 392
F	Marchouch-2016/2017 with no herbicide treatment	Range	34–44	96–106	41–82	1089–3729
		Mean ± SE	39.3 ± 2.0	99.91 ± 1.8	65.4 ± 5.7	2470 ± 499.5
G	Terbol-2015/2016 treated by metribuzin 250 g ai/ha	Range	93–130	165–173	18–77	0–4190
		Mean ± SE	103.6 ± 4.4	168.8 ± 2.3	51.8 ± 8.2	1720 ± 665
H	Terbol-2015/2016 treated by imazethapyr 75 g ai/ha	Range	93–131	165–173	33.0–76.7	57–3689
		Mean ± SE	102.8 ± 3.0	170.1 ± 2.75	57.1 ± 7.3	1439 ± 739.8
I	Terbol-2015/16 with no herbicide treatment	Range	93–122	165–171	39.3–93	352–4184
		Mean ± SE	98.8 ± 1.6	166.1 ± 1.97	68.5 ± 7.3	2313 ± 474
J	Terbol-2018/2019 treated metribuzin 250 g ai/ha	Range	99–130	175–183	49–105	321.2–4782
		Mean ± SE	107.1 ± 2.1	178.7 ± 1.53	79.7 ± 7.98	2423 ± 745.3
K	Terbol-2018/2019 with Imazethapyr 75 g ai/ha	Range	99–130	175–185	48–103	781–5369
		Mean ± SE	106.8 ± 2.3	175 ± 1.33	72.3 ± 8.3	2757 ± 679
L	Terbol-2018/2019 with no herbicide treatment	Range	93–130	175–183	52–113	912–7788
		Mean ± SE	106 ± 2.1	176.8 ± 0.8	83.5 ± 7.6	3424 ± 1173

SE. standard error, DFLR days to 50% flowering after sowing, DMAT days to 80% maturity, PLHT plant height, GY yield, ND. no data.

**Table 3:** Wald statistics performed estimates for detecting differences among faba bean genotypes and narrow sense heritability of plant height and grain yield across environments.

Environment	Environment Characteristics	df	PLHT	*h^2^*_PLHT	GY	*h^2^*_GY
A	Marchouch 2014/2015 treated by metribuzin 250 g ai/ha	36	3755.1 ***	0.95	385.6 ***	0.50
B	Marchouch 2014/2015 treated by imazethapyr 75 g ai/ha	36	99.1 ***	0.50	107.1 ***	0.50
C	Marchouch 2014/2015 with no herbicide treatment	36	203.4 ***	0.50	62.7 **	0.50
D	Marchouch-2016/2017 treated by metribuzin 250 g ai/ha	36	64.9 **	0.03	69.8 *	0.00
E	Marchouch-2016/2017 treated by imazethapyr 75 g ai/ha	36	46.3	0.05	82.1 *	0.00
F	Marchouch-2016/2017 with no herbicide treatment	36	82.1 *	0.32	83.5 *	0.00
G	Terbol-2015/2016 treated by metribuzin 250 g ai/ha	36	149.7 ***	0.18	170.5 ***	0.01
H	Terbol-2015/2016 treated by imazethapyr 75 g ai/ha	36	95.0 ***	0.14	89.7 ***	0.06
I	Terbol-2015/2016 with no herbicide treatment	36	135.0 ***	0.01	352.5 ***	0.12
J	Terbol-2018/2019 treated metribuzin 250 g ai/ha	36	74.9 *	0.01	139.2 ***	0.00
K	Terbol-2018/2019 with Imazethapyr 75 g ai/ha	36	97.9 ***	0.01	130.7 ***	0.24
L	Terbol-2018/2019 with no herbicide treatment	36	160.1 ***	0.02	74.4 *	0.00

Df. degree of freedom, PLHT plant height, h^2^_PLHT narrow sense heritability of plant height, GY grain yield, h^2^_GY narrow sense heritability of grain yield, GE Genotype × Environment interaction, ND. no data, * *p* < 0.05, ** *p* < 0.01, *** *p* < 0.001.

**Table 4:** Cultivar superiority index, static stability, Wricke’s eco-valence and Finlay and Wilkinson values for plant height of faba bean accessions evaluated in 12 different environments. Values marked in bold belong to the 10 most stable accessions, while values presented between brackets reflect their ranking.

Accession	Accession Number	Cultivar Superiority	Static Stability	Wricke’s Eco-valence	Finlay- Wilkinson
IG11561	1	331.9(23)	189.4(19)	519.8(14)	0.9074(16)
IG12110	2	**170.6(4)**	189.4(18)	515.7(13)	0.9941(18)
VF283	3	351.7(26)	218.2(23)	750.1(24)	1.1284(27)
IG13906	4	**199.2(6)**	155.3(12)	764.4(25)	0.8296(12)
IG74363	5	**157.7(3)**	289.9(31)	834.9(27)	1.2719(32)
IG13530	6	342.3(25)	207.5(21)	**201.2(2)**	1.2292(30)
IG13547	7	**216.6(10)**	238.5(28)	620.5(18)	1.0971(24)
VF513	8	399.6(28)	**143(10)**	664.3(20)	0.9026(15)
VF522	9	429.5(31)	206.1(20)	912.3(30)	1.0141(19)
IG11742	10	381.7(27)	231.9(26)	498.4(11)	1.142(28)
IG12659	11	646.1(36)	**120.5(6)**	1312.2(33)	**0.5057(1)**
VF419	12	266.6(17)	161.4(14)	**360.3(5)**	0.9258(17)
IG104039	13	401.7(29)	563.2(36)	2596.2(37)	1.7049(37)
FB2648	14	327.4(21)	270.4(30)	557.2(16)	1.2729(33)
FB2528	15	402.1(30)	157.6(13)	514.3(12)	0.8565(14)
FB2601	16	**212.1(8)**	**104.1(1)**	540.6(15)	**0.6932(6)**
IG104374	17	**155.1(2)**	207.8(22)	**368.3(6)**	1.1143(25)
IG104421	18	**216.4(9)**	174.5(16)	**271.8(3)**	1.0751(22)
IG106453	19	**177(5)**	162.2(15)	641.8(19)	0.8476(13)
Flip 86–98FB	20	**199.6(7)**	**129.9(8)**	**298.8(4)**	1.0777(23)
IFB1216	21	220.4(11)	227.5(25)	672.2(21)	1.1187(26)
IG11527	22	328.2(22)	**118.3(5)**	742.4(23)	**0.7(7)**
VF845	23	339.8(24)	**139(9)**	**181.3(1)**	1.0289(20)
IG99419	24	326.1(20)	178.8(17)	831.6(26)	0.7987(11)
VF324	25	284.5(19)	225.1(24)	905.8(29)	1.0561(21)
VF339	26	557.5(34)	**125.1(7)**	471.9(9)	**0.6844(5)**
VF963	27	227.3(13)	-	**477.7(10)**	**0.7321(8)**
IG13945	28	**84.7(1)**	390.2(35)	2204(36)	1.249(31)
IG14196	29	447.2(33)	357.2(33)	1382.4(34)	1.3134(34)
FB1720	30	565.3(35)	**107.3(2)**	680.9(22)	**0.641(3)**
IG13008	31	263.8(16)	379.8(34)	1229.9(31)	1.5122(36)
IG103043	32	717.7(37)	263.8(29)	2035.4(35)	**0.7726(10)**
IG70622	33	272.5(18)	**114(4)**	**437.8(8)**	**0.7637(9)**
VF545	34	441.5(32)	143.4(11)	1299.7(32)	**0.5994(2)**
IG12983	35	263.7(15)	**112.7(3)**	599.3(17)	**0.6791(4)**
VF810	36	222.1(12)	233.7(27)	**413.1(7)**	1.1556(29)
VF260	37	231.4(14)	351.6(32)	839.4(28)	1.4862(35)

**Table 5:** Correlation coefficients between the stability parameters of the 37 faba bean accessions tested across 12 environments.

Trait	Method	Cultivar Superiority	Finlay and Wilkinson	Static Stability
**PLHT**	Finlay and Wilkinson	−0.4 *	-	-
	Static stability	−0.1	0.9 ***	-
	Wricke’s eco-valence	0.3	0.3	0.7 ***
**GY**	Finlay and Wilkinson	−0.3	-	-
	Static stability	−0.6 ***	0.2	-
	Wricke’s eco-valence	−0.3	0.0	0.6 ***

PLHT plant height; GY grain yield; * significant at the 0.05 probability level.; *** significant at the 0.001 probability level.

**Table 6:** Cultivar superiority index, static stability, Wricke’s eco-valence and Finlay and Wilkinson values for the grain yield of faba bean accessions evaluated in 12 different environments. Values marked in bold belong to the 10 most stable accessions, while values presented between brackets reflect their ranking.

Accession	Accession Number	Cultivar Superiority	Static Stability	Wricke’s Eco-valence	Finlay- Wilkinson
IG11561	1	**22,485(9)**	8927(13)	**29,797(3)**	0.3013(17)
IG12110	2	28,697(15)	12,597(19)	**38,689(6)**	0.2918(23)
VF283	3	48,317(33)	**3891(5)**	**36,277(5)**	0.2909(28)
IG13906	4	26,485(14)	13,087(22)	73,390(22)	0.2973(30)
IG74363	5	38,191(26)	13,269(23)	70,087(19)	**0.2864(5)**
IG13530	6	31,653(20)	13,365(24)	69,391(18)	0.2972(27)
IG13547	7	**14,591(2)**	16,564(28)	88,280(27)	0.3218(21)
VF513	8	**22,008(8)**	22,278(31)	126,327(33)	0.2951(29)
VF522	9	33,940(23)	9839(16)	80,135(24)	0.2838(22)
IG11742	10	48,025(32)	12,793(20)	62,918(14)	0.3075(11)
IG12659	11	41,996(29)	8638(12)	128,754(34)	0.2864(34)
VF419	12	50,741(34)	**2913(2)**	**28,269(2)**	0.2863(16)
IG104039	13	43,884(30)	9326(14)	135,959(35)	0.3008(24)
FB2648	14	24,007(11)	24,953(34)	119,856(31)	0.2838(35)
FB2528	15	40,875(28)	**2728(1)**	57,220(12)	**0.3219(3)**
FB2601	16	56,023(36)	**3523(3)**	106,197(30)	**0.2833(10)**
IG104374	17	31,852(21)	15,414(27)	90,573(28)	**0.3029(2)**
IG104421	18	60,697(37)	**5081(6)**	63,444(15)	0.3014(32)
IG106453	19	**20,382(5)**	12,874(21)	72,541(21)	**0.2953(7)**
Flip 86-98FB	20	**20,704(6)**	13,909(25)	**49,157(9)**	0.2921(20)
IFB1216	21	**17,744(3)**	16,801(29)	91,972(29)	0.2975(31)
IG11527	22	25,508(13)	**6357(9)**	**34,188(4)**	**0.2908(9)**
VF845	23	**4164(1)**	23,917(33)	255,879(37)	0.2882(26)
IG99419	24	30,080(18)	19,235(30)	82,221(25)	0.2918(13)
VF324	25	24,172(12)	**5996(8)**	70,922(20)	0.3094(14)
VF339	26	28,852(17)	8030(11)	**46,802(7)**	0.2951(15)
VF963	27	**20,374(4)**	ND	73,431(23)	0.284(12)
IG13945	28	28,731(16)	**7384(10)**	58,930(13)	0.2695(25)
IG14196	29	31,459(19)	15,412(26)	66,326(17)	0.2951(33)
FB1720	30	40,017(27)	10,146(17)	123,054(32)	**0.3219(8)**
IG13008	31	31,970(22)	23,600(32)	144,645(36)	**0.2974(4)**
IG103043	32	44,948(31)	ND	**49,900(10)**	**0.2833(1)**
IG70622	33	37,920(25)	9628(15)	55,610(11)	0.286(37)
VF545	34	**23,971(10)**	ND	64,208(16)	0.2917(19)
IG12983	35	**21,023(7)**	10,532(18)	**46,866(8)**	0.2908(18)
VF810	36	36,277(24)	**5569(7)**	**15,481(1)**	**0.2953(6)**
VF260	37	54,013(35)	**3550(4)**	85,871(26)	0.1424(36)

**Table 7:** Details of different environments where the faba bean accessions were tested.

Environment Symbol	Environment (Site-Season-Treatment Details)	Rainfall (mm)	Supplemental Irrigation (mm)	Air Temperature (°C)		
				Average	Average Min	Average Max
A	Marchouch-2014/2015 treated by metribuzin 250 g ai/ha	291.4	0	13.12	5.61	23.64
B	Marchouch-2014/2015 treated by imazethapyr 75 g ai/ha		0			
C	Marchouch-2014/2015 with no herbicide treatment		0			
D	Marchouch-2016/2017 treated by metribuzin 250 g ai/ha	211	0	14.05	−2.4	42.99
E	Marchouch-2016/2017 treated by imazethapyr 75 g ai/ha		0			
F	Marchouch-2016/2017 with no herbicide treatment		0			
G	Terbol-2015/2016 treated by metribuzin 250 g ai/ha	343	30	11.5	−0.44	24.62
H	Terbol-2015/2016 treated by imazethapyr 75 g ai/ha		30			
I	Terbol-2015/2016 with no herbicide treatment		30			
J	Terbol-2018/2019 treated metribuzin 250 g ai/ha	810.2	0	11.7	−0.28	32.3
K	Terbol-2018/2019 with Imazethapyr 75 g ai/ha		0			
L	Terbol-2018/2019 with no herbicide treatment		0			

Min-Minimal temperature during cropping season; Max-Maximal temperature during cropping season.

## Data Availability

Data is contained within the article and Supplementary Material.

## Supplementary Materials

The following are available online at https://www.mdpi.com/article/10.3390/plants11030251/s1, Table S1: Faba bean accessions with different degree of tolerance to Metribuzin and Imazethapyr used in the present study.
