# Supplementary material for: Adaptability and Stability of Faba Bean (Vicia faba L.) Accessions under Diverse Environments and Herbicide Treatments
Source: Plants (Basel). 2022 Jan 19;11(3):251. doi: 10.3390/plants11030251 (PMC8839948; doi:10.3390/plants11030251)
Supplement: Supplementary file 1 [file plants-11-00251-s001.zip › plants-1502151-supplementary.pdf]

plants

*Supplementary Material*

**Table S1.** Faba bean accessions with different degree of tolerance to Metribuzin and Imazethapyr used in the present study.

| Accession Number | Accession Name | Origin | Response to Metribuzin @ 250 g ai/ha | Response to Imazethapyr @ 75 g ai/ha |
|---|---|---|---|---|
| 1 | IG11561 | Algeria | T | T |
| 2 | IG12110 | Algeria | T | T |
| 3 | VF283 | Bulgaria | T | MT |
| 4 | IG13906 | Canada | MT | T |
| 5 | IG74363 | Canada | T | MT |
| 6 | IG13530 | Cyprus | MT | T |
| 7 | IG13547 | Cyprus | T | MT |
| 8 | VF513 | Egypt | MT | T |
| 9 | VF522 | Egypt | T | T |
| 10 | IG11742 | Ethiopia | T | MT |
| 11 | IG12659 | Ethiopia | MT | MT |
| 12 | VF419 | Ethiopia | MT | MT |
| 13 | IG104039 | Ethiopia | T | T |
| 14 | FB2648 | Ethiopia | T | T |
| 15 | FB2528 | France | T | T |
| 16 | FB2601 | France | T | T |
| 17 | IG104374 | Germany | MT | T |
| 18 | IG104421 | Germany | T | T |
| 19 | IG106453 | Greece | MT | T |
| 20 | Flip 86-98FB | Lebanon | MT | T |
| 21 | FB1216 | Netherlands | MT | T |
| 22 | IG11527 | Pakistan | MT | MT |
| 23 | VF845 | Peru | MT | T |
| 24 | IG99419 | Portugal | MT | T |
| 25 | VF324 | Russia | MT | T |
| 26 | VF339 | Russia | MT | T |
| 27 | VF963 | Spain | T | T |
| 28 | IG13945 | Sudan | MT | T |
| 29 | IG14196 | Sudan | MT | T |
| 30 | FB1720 | Switzerland | MT | T |
| 31 | IG13008 | Syria | MT | T |
| 32 | IG103043 | Syria | MT | MT |
| 33 | IG70622 | Syria | T | T |
| 34 | VF545 | Tunisia | T | MT |
| 35 | IG12983 | Tunisia | T | T |
| 36 | VF810 | United Kingdom | MT | T |
| 37 | VF260 | Unknown | T | MT |

S susceptible, R resistant, MT moderately tolerant, according to Abou-Khater et al., 2021 and ICARDA, unpublished data.
